# Heat-Processed Soybean Germ Extract and *Lactobacillus gasseri* NK109 Supplementation Reduce LPS-Induced Cognitive Impairment and Colitis in Mice

**DOI:** 10.3390/nu16162736

**Published:** 2024-08-16

**Authors:** Soo-Won Yun, Dong-Yun Lee, Hee-Seo Park, Dong-Hyun Kim

**Affiliations:** Neurobiota Research Center, Department of Pharmacy, Kyung Hee University, 26, Kyungheedae-ro, Dongdaemun-gu, Seoul 02447, Republic of Korea; ysw6923@naver.com (S.-W.Y.); dongyun8246@naver.com (D.-Y.L.); xlvksl1997@khu.ac.kr (H.-S.P.)

**Keywords:** soybean embryo ethanol extract, *Lactobacillus gasseri* NK109, cognitive impairment, colitis, lipopolysaccharide

## Abstract

Soybean alleviates cognitive impairment. In our preparatory experiment, we found that dry-heat (90 °C for 30 min)-processed soybean embryo ethanol extract (hSE) most potently suppressed lipopolysaccharide (LPS)-induced tumor necrosis factor (TNF)-α expression in BV2 cells among dry-heat-, steaming-, and oil exclusion-processed soybean embryo ethanol extracts (SEs). Heat processing increased the absorbable soyasaponin Bb content of SE. Therefore, we investigated whether hSE and its supplement could mitigate LPS-impaired cognitive function in mice. Among dry-heat-, steaming-, and oil exclusion-processed SEs, hSE mitigated LPS-impaired cognitive function more than parental SE. hSE potently upregulated LPS-suppressed brain-derived neurotropic factor (BDNF) expression in the hippocampus, while LPS-induced TNF-α and IL-1β expression in the hippocampus and colon were downregulated. *Lactobacillus gasseri* NK109 additively increased the cognitive function-enhancing activity of hSE in mice with LPS-induced cognitive impairment as follows: the hSE and NK109 mix potently increased cognitive function and hippocampal BDNF expression and BDNF-positive neuron cell numbers and decreased TNF-α expression and NF-κB-positive cell numbers in the hippocampus and colon. These findings suggest that hSE and its supplement may decrease colitis and neuroinflammation by suppressing NF-κB activation and inducing BDNF expression, resulting in the attenuation of cognitive impairment.

## 1. Introduction

Dementia is a strongly age-related syndrome leading to a deterioration in cognitive and behavioral functions [1]. The most common type of dementia (60–70%) is Alzheimer’s disease, followed by vascular dementia [1,2]. Its pathogeneses have been reported to be related to neuronal inflammation, amyloid-β (Aβ) plaque, and fibrillary tangles in the nervous system [3,4,5]. In particular, neuronal inflammation and Aβ aggregation can be accelerated by gut microbiota dysbiosis, which increases gut bacterial toxins such as lipopolysaccharide (LPS). Excessive exposure to gut bacterial LPS causes systemic inflammation, including neuroinflammation, in rodents by activating toll-like receptor 4-mediated NF-κB signaling [5].

Exposure to pathogen infections, stressors, and antibiotics can cause gut microbiota dysbiosis [6,7]. The gut microbiota dysbiosis induce psychiatric disorders such as dementia and depression through the NF-κB signaling-suppressed brain-derived neurotropic factor (BDNF) expression [8,9]. However, *Lactobacillus gasseri* NK109, an interleukin (IL)-1β expression-suppressing bacterium isolated from human gut microbiota, alleviates *Escherichia coli*-impaired cognitive function in germ-free and specific pathogen-free mice by modulating gut microbiota [10]. NK109 also mitigates cognitive function in aged mice [11]. *Lactobacillus mucosae* NK41 and *Bifidobacterium longum* NK46 also alleviate *Escherichia coli* K1-impaired cognitive function in mice by upregulating BDNF expression [12,13]. Therefore, upregulating NF-κB activation-suppressed BDNF expression may be useful for the improvement of cognitive impairment.

Soybean (glycine max) and its embryo contain carbohydrates, proteins, fats, and bioactive phytochemicals such as isoflavones and soyasaponins [14,15,16,17]. The soybean embryo exhibits the high contents of phytochemicals including isoflavones and soyasaponins compared to soybean [16,17]. Of these phytochemicals, soyasaponins Ab and Bb (I), daidzin, and genistin exhibit anti-inflammatory, neuroprotective, and osteoporosis-ameliorating effects [17,18,19,20]. Soyasaponins Ab and Bb alleviate LPS-induced cognitive impairment and inflammation in vivo by inhibiting NF-κB signaling [20,21]. The fermentation of defatted soybean (SD) by *Lactobacillus plantarum* C29 increases cognitive impairment-ameliorating activities in vivo [22]. NK109 supplemented with soybean embryo ethanol extract (SE) also alleviates cognitive impairment in mice [11]. However, studies on the biological effects of soybean embryo and its supplements remain limited.

Therefore, to investigate the action mechanism of SE against cognitive impairment and increase the cognitive impairment-ameliorating activity of SE, we processed SE using dry-heat, steaming, and hexane-extraction (oil) exclusion and examined their effects on cognitive function and neuroinflammation in mice with LPS-induced cognitive impairment (LCI). Furthermore, we investigated the effects of SE and hSE with NK109 on LCI in mice.

## 2. Materials and Methods

### 2.1. Materials

LPS, DMEM, and 4′,6-diamidino-2-phenylindole, dilactate (DAPI) were purchased from Sigma (St Louis, MI, USA).

### 2.2. Culture of NK109

NK109 was cultured in MRS broth (BD) at 37 °C for 24 h, centrifuged, and freeze-dried [11].

### 2.3. Preparation of SE and Its Supplement

First, SE (10 g, contained 0.2% soyasaponin Bb, Mirae Biotech, Seoul, Republic of Korea) was dry-heated at 90 °C for 30 min with shaking. It was named heated SE (hSE). Second, SE was steamed at 90 °C for 30 min and freeze-dried. It was named steamed SE (sSE). Third, SE was soaked with hexane three times for 24 h. The insoluble precipitate was dried and used as organic solvent extraction-excluded SE (oSE).

### 2.4. Determination of the Soayasopnin Bb Content in SE and hSE

The content of soyasaponin Bb in SE and hSE was analyzed by high-performance liquid chromatography, as previously reported [21].

### 2.5. Cultures of BV-2 Cells

BV2 cells (Korea Cell Line Bank, Seoul, Republic of Korea) were cultured in DMEM containing 1% antibiotic–antimycotic and 5% fetal bovine serum at 37 °C, as previously reported [23]. BV-2 cells (1 × 10^6^ cells/mL) were incubated with test agents in the absence or presence of LPS (100 ng/mL, Sigma, St Louis, MI, USA) for 20 h. In the supernatant, TNF-α and IL-10 levels were determined using enzyme-linked immunosorbent assay (ELISA) kits (Ebioscience, Atlanta, GA, USA).

### 2.6. Animals

Male C57BL/6 mice (6 weeks-old, 19–21 g) were purchased from the Samtacho animal breeding center and adapted for one week before the usage of experiments, kept under the controlled condition (temperature, 22 °C ± 1 °C; humidity, 50% ± 10%; light cycle, 12 h [07:00–19:00]) and fed with food and water under ad libitum conditions. All experiments were approved by the University Laboratory Animal Care and Use Committee (IACC No., KHUASP(SE)-21306, 15 July 2021) and ethically conducted in the accordance with the University Ethical Policies and Guidelines for the Care and Use of Laboratory Animals and ARRIVE guideline [24].

### 2.7. Cognitive Function-Impaired Mouse Preparation

Mice with LCI were prepared, as previously reported [19]. Each group consisted of seven mice.

First, to investigate the effects of processed SEs against cognitive impairment, mice were separated into six groups (NC, LP, SE, sSE, hSE, and oSE) and (except NC) intraperitoneally injected with LPS (10 μg/kg/day, dissolved in saline) for 5 days. The day after the final LPS injection, test agents (LP, vehicle alone; SE, 50 mg/kg SE; sSE, 50 mg/kg sSE; hSE, 50 mg/kg hSE; and oSE, 50 mg/kg oSE) were orally gavaged daily for 5 days. NC was treated with saline instead of LPS and test agents.

Second, to confirm the effects of SE and hSE on cognitive impairment, mice were separated into four groups (NC, LP, SE, hSE). Mice were intraperitoneally injected with LPS daily for 5 days. The day after the final LPS injection, test agents (LP, vehicle; SE, 50 mg/kg SE; hSE, 50 mg/kg hSE) were orally gavaged daily for 5 days. NC received saline instead of LPS/test agents.

Third, to confirm the effect of *Lactobacillus gasseri* NK109 on cognitive impairment, mice were separated into three groups (NC, LP, NK). Mice were intraperitoneally injected with LPS for 5 days. The day after the final LPS injection, test agents (LP, vehicle; NK, 5 × 10^8^ CFU/mouse NK109) were orally gavaged daily for 5 days. NC received saline instead of LPS and test agents.

Fourth, to investigate the effects of SE and NK109 mix (SN) and hSE and NK109 mix (hSN) on cognitive impairment, mice were separated into four groups (NC, LP, SN, and hSN) and intraperitoneally injected with LPS for 5 days. The day after the final injection of LPS, test agents (LP, vehicle; SN, 50 mg/kg SE plus 5 × 10^8^ CFU/mouse NK109; hSN, 50 mg/kg hSE plus 5 × 10^8^ CFU/mouse NK109) were orally gavaged once a day for 5 days. NC received saline instead of LPS and test agents.

The day after the final treatment with test agents, cognitive behaviors were measured in the Y-maze task (YMT) and novel object recognition tests (NORTs). Mice were euthanized by the inhalation of CO_2_ and then sacrificed by cervical dislocation. The sera, hippocampus, and colon were collected and stored at −80 °C for the assay of biochemical markers. Mice were transcardiacally perfused with 4% paraformaldehyde and their brain and colon tissues post-fixed with paraformaldehyde, cytoprotected in sucrose solution, and frozen [20].

### 2.8. Behavioral Tasks

The cognitive function was measured using the YMT in the apparatus with a three-arm horizontal maze (40 [length] × 3 [width] × 12 cm [height]) and NORT in a black rectangular open field apparatus (45 × 45 × 45 cm), as previously reported [10,19].

### 2.9. Immunoblotting and ELISA

Hippocampus and colon tissues were lysed in radio immunoprecipitation assay lysis buffer (Pierce, Rockford, IL, USA) and centrifuged (10,000× *g*, 4 °C, 10 min). The following are the resulting supernatants that were used for the analysis of ELISA: BDNF, IL-1β, IL-10, TNF-α. Myeloperoxidase expression levels were assayed using commercial ELISA kits (Ebioscience, Atlanta, GA, USA) [12].

### 2.10. Immunofluorescence Staining

Immunofluorescence staining for the hippocampus and colon were performed, as previously reported [12,25].

### 2.11. Statistical Analysis

Data are indicated as mean ± S.D. using GraphPad Prism 9 (GraphPad Software, Inc., San Diego, CA, USA). The significance was analyzed using one-way ANOVA followed by Duncan’s multiple range test (*p* < 0.05).

## 3. Results

### 3.1. SEs Decreased LPS-Induced TNF-α Expression in BV2 Cells

**Figure 1:**
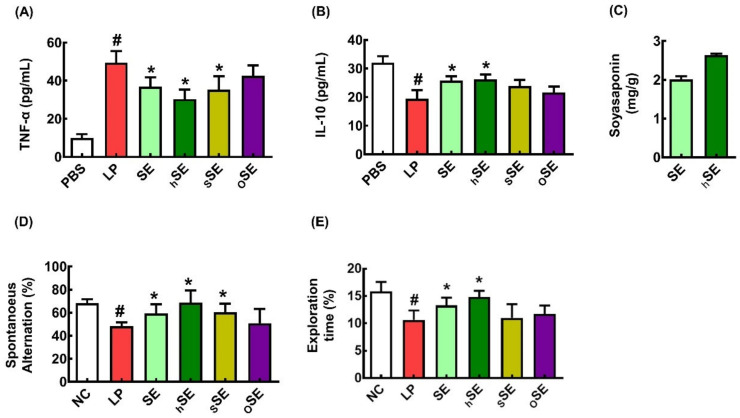
The effect of SEs on LPS-induced TNF-α and IL-10 expression in BV2 cells and on LCI in mice. Effect on TNF-α expression (**A**) and IL-10 expression (**B**) in LPS-stimulated BV-2 cells. NC, vehicle (PBS); hSE, dry-heated SE + LPS; sSE, steamed SE + LPS; sSE, steamed SE + LPS; oSE, hexane-treated + LPS. *n* = 4. ^#^ *p* < 0.05 vs. NC. * *p* < 0.05 vs. LP. (**C**) The content of soyasaponin Bb in hSE and SE. *n* = 4. * *p* < 0.05 vs. SE. Effects on spontaneous alternation in YMT (**D**) and recognition in NORT (**E**). NC, vehicle (saline); LP, vehicle + LPS; SE, 50 mg/kg SE + LPS; sSE, 50 mg/kg sSE + LPS; hSE, 50 mg/kg hSE + LPS; and oSE, 50 mg/kg oSG + LPS. *n* = 7. ^#^ *p* < 0.05 vs. NC. * *p* < 0.05 vs. LP.

To investigate the effect of food processing on the anti-inflammatory effect of SE, we examined the effects of processed SEs (hSE, dry-heat-processed SE; sSE, steaming-processed SE; oSE, hexane extrusion-processed SE) on LPS-induced TNF-α and IL-10 expression in BV2 cells (Figure 1A,B). The LPS stimulation increased the expression of TNF-α in BV2 cells. However, processed SEs strongly reduced the LPS-induced expression of TNF-α. Of these, hSE most potently decreased the TNF-α to IL-10 expression ratio, followed by SE. Interestingly, heat processing increased the content of soyasaponin Bb as follows: the soyasaponin Bb content of SE and hSE was 2.00 ± 0.01 mg/g and 2.64 ± 0.03 mg/g, respectively (Figure 1C).

Next, we examined the effects of SEs on LCI in mice. Of these, SE and hSE (50 mg/kg) significantly mitigated LCI-like behavior in the YMT and NORT (Figure 1D,E). The sSE and oSE weakly, but not significantly, mitigated LCI-like behaviors.

### 3.2. SE and hSE Alleviated LCI in Mice

**Figure 2:**
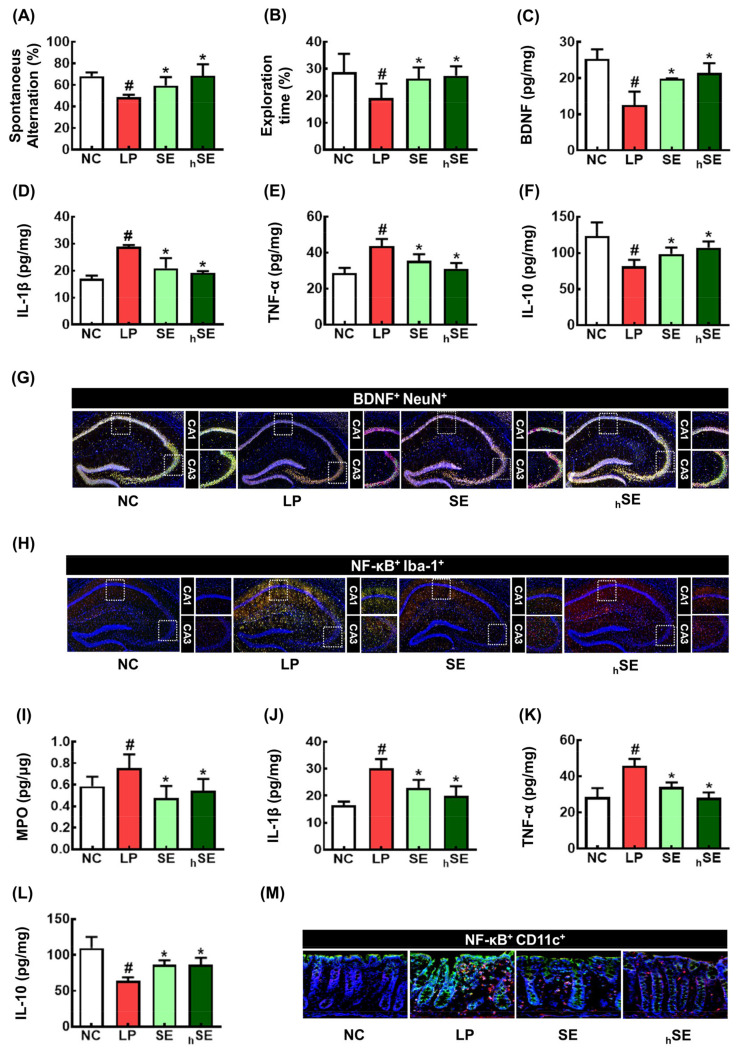
The effects of SE and hSE on LCI-like behavior, neuroinflammation, and gut inflammation in mice. (**A**) Effects on spontaneous alternation in YMT (**A**) and recognition in NORT (**B**). Effects on hippocampal BDNF (**C**), IL-1β (**D**), TNF-α (**E**), and IL-10 expression (**F**), assessed by ELISA. Effects on BDNF^+^NeuN^+^ cell (**G**) and NF-κB^+^Iba1^+^ cell numbers (**H**), assessed by a confocal microscope. Effects on myeloperoxidase (MPO) (**I**), IL-1β (**J**), TNF-α (**K**), and IL-10 (**L**) expression and NF-κB^+^Iba1^+^ cell numbers (**M**) in the colon. NC, vehicle; LP, vehicle + LPS; SE, 50 mg/kg SE + LPS; hSE, 50 mg/kg hSE + LPS. *n* = 7. ^#^ *p* < 0.05 vs. NC. * *p* < 0.05 vs. LP.

To confirm the effects of SE and hSE against cognitive impairment, we examined their effects on LCI in mice (Figure 2). The LPS treatment significantly decreased spontaneous alternation in the YMT to 71.4% of NC (Figure 2A). However, orally administered SE and hSE alleviated LCI in the YMT by restoring it to 83.8% and 99.5% of NC, respectively. However, treatment with the vehicle, LPS, SE, and hSE did not significantly affect average values of the arm entry numbers in the YMT, suggesting that that their treatments did not affect general locomotor activity, as previously reported [26]. The LPS exposure also reduced exploration in the NORT to 56.7% of NC (Figure 2B). Orally administered SE and hSE increased LPS-decreased exploration time to 81.4% and 89.5 of NC, respectively. The cognitive impairment-ameliorating effect of hSE was more potent than that of SE.

The LPS exposure increased the expression of TNF-α and IL-1β in the hippocampus, while the expression of IL-10 decreased (Figure 2C–H). However, orally administered SE or hSE significantly downregulated the LPS-induced expression of TNF-α and IL-1β and the number of NF-κB^+^Iba1^+^ cells in the hippocampus. Their treatments upregulated the LPS-suppressed expression of BDNF and IL-10 and the number of BDNF^+^NeuN^+^ cells.

Furthermore, LPS exposure significantly increased myeloperoxidase, TNF-α, and IL-1β expression and decreased IL-10 expression in the colon (Figure 2I–M). The inflammatory biomarker expression was downregulated by treatment with SE or hSE, resulting in the attenuation of colitis. The anti-inflammatory effect of hSE in the colon was more potent than that of SE.

### 3.3. NK109 Decreased LPS-Induced TNF-a Expression in BV2 Cells

**Figure 3:**
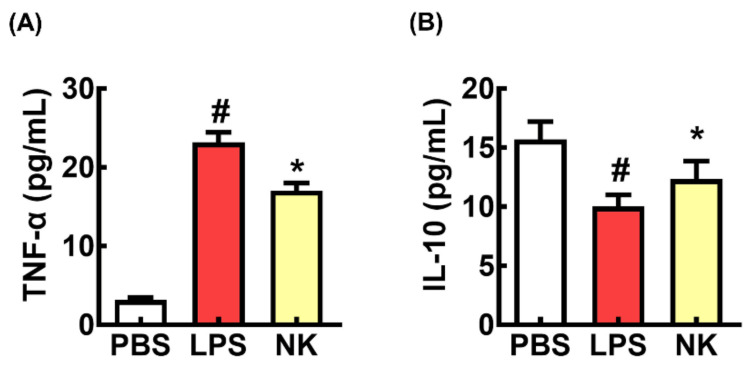
The effect of NK109 on LPS-induced TNF-α (**A**) and IL-10 expression (**B**) in BV2 cells. PBS, PBS alone; LPS, vehicle + LPS; NK NK109 (1 × 10^5^ CFU/mL) + LPS. *n* = 4. ^#^ *p* < 0.05 vs. PBS. * *p* < 0.05 vs. LPS.

Next, the effect of NK109 on LPS-induced TNF-α expression was measured in BV2 cells (Figure 3). The LPS stimulation upregulated the expression of TNF-α and downregulated the expression of IL-10. However, NK109 treatment downregulated the LPS-induced TNF-α to IL-10 expression ratio.

### 3.4. NK109 Alleviated LCI in Mice

**Figure 4:**
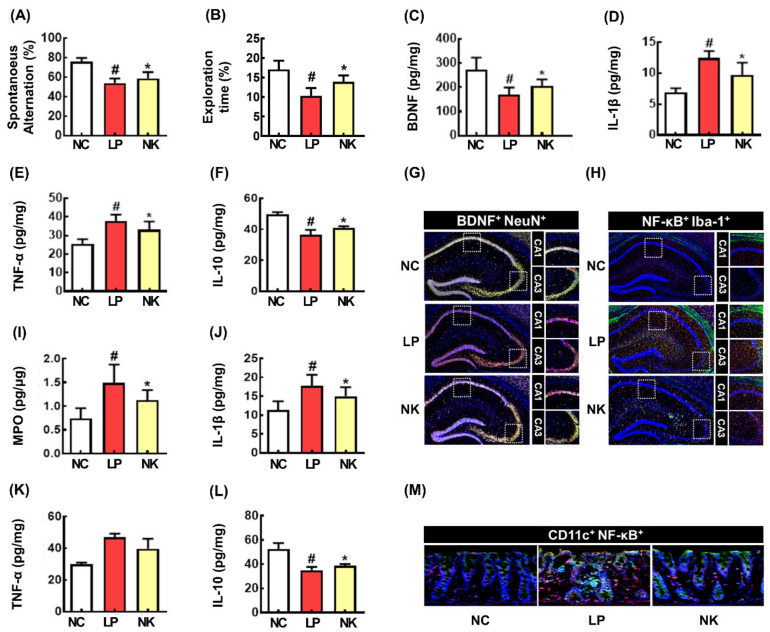
The effect of NK109 on the LCI in mice. (**A**) Effect on spontaneous alternation in the YMT (**A**) and recognition in NORT (**B**). Effect on hippocampal BDNF (**C**), IL-1β (**D**), TNF-α (**E**), and IL-10 expression (**F**). Effect on BDNF^+^NeuN^+^ cell (**G**) and NF-κB^+^Iba1^+^ cell numbers (**H**). Effect on colonic myeloperoxidase (MPO) (**I**), IL-1β (**J**), TNF-α (**K**), and IL-10 (**L**) expression and NF-κB^+^Iba1^+^ cell numbers (**M**). NC, vehicle; LP, vehicle + LPS; NK, 5 × 10^8^ CFU/mouse NK109 + LPS. *n* = 7. ^#^ *p* < 0.05 vs. NC. * *p* < 0.05 vs. LP.

Next, the effects of NK109 on LCI were investigated in mice (Figure 4). The LPS injection significantly impaired spontaneous alternation in the YMT to 69.2% of NC (Figure 4A). However, SE treatment weakly alleviated LCI-like behavior to 77.5% of NC. LPS exposure also reduced exploration in the NORT to 60.6% of NC (Figure 4B). NK109 significantly increased LPS-suppressed exploration to 80.67% of NC.

NK109 significantly decreased the expression of IL-1β and TNF-α and the number of NF-κB^+^Iba1^+^ cells and increased the LPS-suppressed expression of BDNF and IL-10 and the number of BDNF^+^NeuN^+^ cells in the hippocampus (Figure 4C–H). NK109 also suppressed the LPS-induced expression of colonic myeloperoxidase, TNF-α, and IL-1β expression, resulting in the attenuation of colitis (Figure 4I–M).

### 3.5. The Combined Effects of NK109 with NK109 (SN) and hSE with NK109 (hSN) on LCI and Colitis in Mice

**Figure 5:**
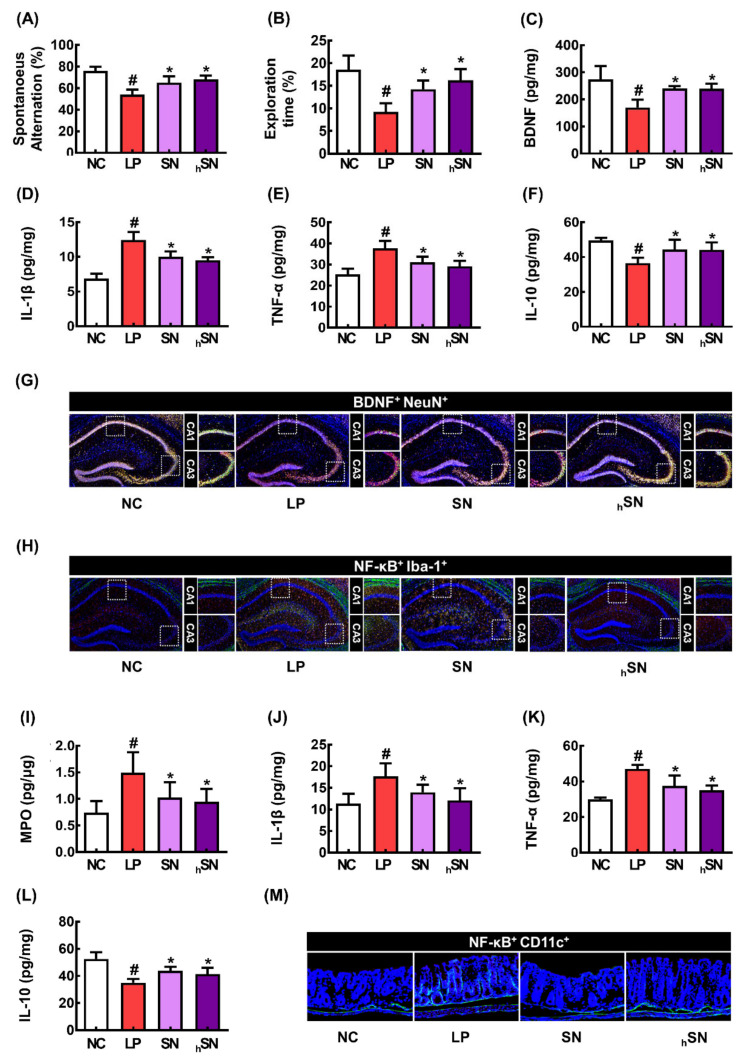
The effects of SN and hSN on LCI in mice. Effects on spontaneous alternation in YMT (**A**) and recognition in NORT (**B**). Effects on hippocampal BDNF (**C**), IL-1β (**D**), TNF-α (**E**), and IL-10 expression (**F**). Effects on BDNF^+^NeuN^+^ cell (**G**) and NF-κB^+^Iba1^+^ cell numbers (**H**). Effects on colonic myeloperoxidase (MPO) (**I**), IL-1β (**J**), TNF-α (**K**), and IL-10 (**L**) expression and NF-κB^+^Iba1^+^ cell numbers (**M**). NC, vehicle; LP, vehicle + LPS; SN, 50 mg/kg SE + 5 × 10^8^ CFU/mouse NK109 + LPS; hSN, 50 mg/kg hSE + 5 × 10^8^ CFU/mouse NK109 + LPS. *n* = 7. ^#^ *p* < 0.05 vs. NC. * *p* < 0.05 vs. LP.

Next, we investigated the effects of SN and hSN on LCI in mice (Figure 5). The SN and hSN significantly alleviated LCI-like behavior in the YMT and NORT (Figure 5A,B). They also suppressed the LPS-induced expression of IL-1β and TNF-α and the number of NF-κB^+^Iba1^+^ cells in the hippocampus, whereas their treatments increased the expression of BDNF and the number of BDNF^+^NeuN^+^ cells (Figure 5C–H). LPS-induced colonic myeloperoxidase and TNF-α expression was decreased by their treatments (Figure 5I–M). The anti-inflammatory efficacy of hSN was more potent than that of SN.

## 4. Discussion

The gut cross-talks with the brain via neural, immune, and endocrine networks [27]. Therefore, gut inflammation, including inflammatory bowel disease, is closely involved in the occurrence of dementia [28]. The suppression of gut inflammation by phytochemicals or probiotics mitigates cognitive impairment and depression [9,18,29]. *Lactobacillus plantarum* C29 mitigates cognitive function and gut inflammation in 5xFAD-transgenic mice [22]. The phytochemicals of soybean such as soyasaponin Ab and Bb and isoflavones have anti-inflammatory and cognitive function-enhancing activities in mice [21,22].

In the present study, SE alleviated LCI-like behavior, downregulated LPS-induced TNF-α and IL-1β levels and the NF-κB-positive immune cell population, and upregulated IL-10 levels in the hippocampus, as shown in the report that discussed how fermented SD and its soyasaponins could alleviate cognitive impairment in rodents by suppressing NF-κB signaling [30,31]. SE also alleviated LPS-induced myeloperoxidase and TNF-α expression in the colon, resulting in the amelioration of colitis. Lee et al. insisted that soyasaponin Bb improved TNBS-induced colitis in mice [30]. Hong et al. insisted that soyasaponin Bb mitigated ibotanic acid-induced learning/memory damage by minimizing neuroinflammation [20]. These results imply that SE can mitigate cognitive impairment through the attenuation of colitis and neuroinflammation by inhibiting NF-κB signaling, in particular the suppression of pro-inflammatory cytokines to anti-inflammatory cytokine expression ratio. Furthermore, SE increased hippocampal BDNF expression and BDNF-positive cells. LPS suppresses BDNF expression by upregulating NF-κB signaling, resulting in cognitive impairment with neuroinflammation [19,32]. BDNF induces long-term potentiation in hippocampal pyramidal neurons [33,34]. These findings imply that SE may mitigate LPS-induced colitis and neuroinflammation by suppressing NF-κB activation and inducing BDNF expression, resulting in the amelioration of cognitive impairment.

Orally administered natural glycosides of saponins such as soyasaponins conjugated with 2,3-dihydro-2,5-dihydroxy-6-methyl-4H-pyran-4-one (DDMP) are not easily absorbed from the gastrointestinal tract to the body due to their hydrophilicities and macromolecules [35,36]. Therefore, to enhance the absorption of hydrophilic and macromolecular natural components, food processes such as heating and fermentation have been developed. Lee et al. reported that the fermentation with *L. plantarum* C29 increased the cognitive impairment-ameliorating activity of SD, the soybean powder [37]. We also found that the addition of NK109 additively increased the cognitive impairment-ameliorating activity of SE in mice [11]. However, the addition of NK109 did not increase the content of absorbable soyasaponins such as soyasaponin Bb. Therefore, in the present study, we processed SE using dry-heating, steaming, and hexane-extraction exclusion. Of these processes, the dry-heating process most potently increased the cognitive impairment-ameliorating effect of SE. In particular, hSE (heat-processed SE) alleviated LCI and suppressed LPS-induced neuroinflammation in mice. hSE also increased LPS-suppressed BDNF expression in the hippocampus. hSE potently suppressed LPS-induced TNF-α expression in microglial BV2 cells. Of the processed SEs, hSE contained the highest content of soyasaponin Bb. Thus, dry-heating process increased the content of bioactive soyasaponin Bb as fermented SD, as previously reported [19]. Many soyasaponins conjugated with DDMP are transformed into DDMP-deconjugated soysaponins such as soyasaponin Bb by alkaline hydrolysis and cooking [38,39]. DDMP-deconjugated soysaponins Ab and Bb alleviate cognitive impairment in mice [20,21]. However, steaming and hexane extraction, which hardly hydrolyze macromolecular soyasaponins, did not significantly increase the cognitive impairment-ameliorating effect of SE and might be transformed to soyasaponin Bb. Therefore, the increased efficacy of hSE may be due to the increase in the content of hydrophobic soyasaponin Bb by the heat process.

In addition, NK109 alleviates *Escherichia coli*-impaired cognitive function in germ-free and specific pathogen-free mice by modulating gut microbiota [10]. NK109 increases cognitive function in aged mice [11]. Moreover, hSN, which is the supplementation of NK109 in hSE, mitigated LCI-like behavior, neuroinflammation, and colitis in mice. The efficacy of hSE was additively increased by the supplementation of NK109. Furthermore, the cognitive impairment-ameliorating activity of hSN was more potent than that of SN (the mixture of non-heated SE and NK109). These results imply that hSE and its supplement can alleviate cognitive impairment by increasing NF-κB activation-suppressed BDNF expression.

## 5. Conclusions

Heat processing may increase the efficacy of SE on cognitive impairment and colitis through the increase in the content of soyasaponin Bb. hSG and its supplement (combined with NK109) may alleviate LPS-induced systemic inflammation by suppressing NF-κB activation and inducing BDNF expression, resulting in the amelioration of cognitive impairment.

## Data Availability

The datasets used and/or analyzed during the current study are available from the corresponding author on reasonable request.
